# Screening 1021 Swedish memory clinic visitors for autoantibody-mediated encephalitis

**DOI:** 10.1007/s00415-026-13953-4

**Published:** 2026-06-20

**Authors:** A. Freitas-Huhtamäki, N. Kleebauer, A. Gardner, J. Lundberg, M. Esbjörnsson, R. Da Silva Rodrigues, P. Waters, M. Scheller-Nissen, M. Blaabjerg, N. Bogdanovic, J. Theorell

**Affiliations:** 1 Center for Infectious Medicine, Department of Medicine Huddinge, Karolinska Institutet, CIM/ANA Futura, Alfred Nobles Allé 8B, 141 52 Stockholm, Sweden; 2 Medical unit of Geriatric Medicine, Theme Inflammation and Aging, Karolinska University Hospital, Stockholm, Sweden; 3 Department of Clinical Sciences, University of Lund, Lund, Sweden; 4 Department of Internal Medicine, Hässleholm Hospital, Hässleholm, Sweden; 5 Medical Unit of Clinical Immunology and Transfusion Medicine, Function Medical Diagnostics, Karolinska University Hospital, Stockholm, Sweden; 6 Department of Medicine Solna, Center for Molecular Medicine, Karolinska Institutet, Stockholm, Sweden; 7 Nuffield Department of Clinical Neurosciences, University of Oxford, Oxford, UK; 8 Department of Clinical Medicine, University of Southern Denmark, Odense, Denmark; 9 Department of Neurology, University Hospital Odense, Odense, Denmark; 10 Medical Unit of Neurology, Theme Heart, Vascular and Neuro, Karolinska University Hospital, Stockholm, Sweden

**Keywords:** Autoimmune encephalitis, Neurodegenerative dementia, Neuroimmunology, Autoantibody detection, Screening

## Abstract

**Introduction:**

A treatable differential diagnosis to neurodegenerative dementia is autoimmune encephalitis (AE). In Sweden, the prevalence of AE varies considerably between regions, suggesting underdiagnosis. We hypothesized that some undetected AE patients would attend Swedish memory clinics.

**Methods:**

We retrospectively screened 1021 individuals attending the Karolinska University Hospital memory clinics for AE autoantibodies. Serum screening was performed with live cell-based assays (CBAs) for antibodies to contactin-associated protein-like 2 (CASPR2), gamma-aminobutyric acid receptor B (GABA_B_R), immunoglobulin superfamily containing LAMP, OBCAM, and neurotrimin family member 5 (IgLON5), leucine-rich glioma-inactivated 1 (LGI1), and n-methyl-d-aspartate receptor (NMDA-R). Positive serum and CSF samples were further tested with fixed CBAs and tissue-based assays.

**Results:**

Eleven patients were antibody-positive in two or more independent tests. When investigating clinical information, three patients had phenotypes suggesting paraneoplastic (PE) or autoimmune encephalitis. These patients had antibodies to either CASPR2 or NMDA-R in serum and CSF and either had CSF-specific electrophoresis IgG-bands or increased Kappa free light-chain-intrathecal fraction. In four patients, available clinical information was insufficient to clarify if the antibodies were related to an ongoing or past encephalitis or lacked relevance, with a further three considered false positive. The final individual had previously been diagnosed with LGI1-AE. When the STAM3mP score, a tool for identifying treatment-responsive rapidly progressive dementia, was used on the seven previously undiagnosed patients with antibodies of possible relevance, six got one point, indicating potential treatment response.

**Conclusion:**

AE is a rare differential diagnosis in our study of memory clinic visitors. Given the low prevalence, we conclude that AE autoantibody testing is not suitable for unselective screening in this setting. Instead, a rigorous clinical evaluation, potentially supported by tools like the STAM3mP score, might acceptably limit the number of missed AE cases among patients suffering from memory impairment, but further studies on pre-screening biomarkers are warranted.

**Supplementary Information:**

The online version contains supplementary material available at 10.1007/s00415-026-13953-4.

## Introduction

Autoimmune encephalitides (AEs) are a group of inflammatory brain disorders where commonly IgG autoantibodies directed against extracellular epitopes on neuronal proteins alter neuronal function [1]. First defined around 20 years ago [2–4], this group is expanding both in clinical presentations and in prevalence with increasing recognition [5, 6]. Depending on which neuronal protein that is targeted, the patients can get a wide spectrum of symptoms, such as seizures, affective lability, psychosis, movement aberrances and autonomic dysregulation [1]. A common feature of most AE syndromes is short-term memory impairment, which tends to increase as the disorder progresses. Complicating the matter, AEs presenting later in life tend to be more subtle and slowly progressing, increasing the risk of a late or missed diagnosis and therefore significant memory impairment [1]. In line with this, Bastiaansen et al. showed in a cohort of 175 autoimmune encephalitis patients presenting after 45 years of age, 38% fulfilled dementia criteria without prominent seizures and 18% were initially suspected of suffering from neurodegenerative dementia [7], with the most prevalent autoantigens being contactin-associated protein-like 2 (CASPR2), leucine-rich, glioma-inactivated 1 (LGI1), gamma-aminobutyric acid receptor B (GABA_B_R), and the n-methyl-d-aspartate receptor (NMDA-R) subunit 1 (GRIN1), the autoantibody target in NMDA-R encephalitis. Furthermore, the same group showed that 7/920 (0.8%) individuals with suspected neurodegenerative dementia had antibodies to either dipeptidyl-deptidase-like protein-6 (DPPX), immunoglobulin superfamily containing LAMP, OBCAM, and neurotrimin family member 5 (IgLON5), NMDA-R or LGI1. Retrospectively, these patients showed signs of AE, such as subacute onset or fluctuating symptoms, but generally lacked overt inflammation or brain magnetic resonance tomography (MRT) abnormalities [8].

Similar to what has been reported elsewhere [5], Sweden seems to have a geographically uneven distribution of AE cases. In a study covering a fifth of the population (~ 2 million people) over 5 years, Kosek and coauthors estimate an annual incidence of 3.3 per million inhabitants per year, with a dominance of AE with 65 kDa glutamic acid decarboxylase (GAD65) or NMDA-R autoantibodies [9]. In contrast, 5-year clinical observations in Hässleholm hospital, Skåne, Sweden, a smaller hospital with considerable AE experience, suggest higher annual incidence rates of up to 40 per million inhabitants per year in the catchment area, or 12 times higher than in the published prevalence study. The commonest autoantibody targets in this group are LGI1, CASPR2, as well as paraneoplastic antigens, i.e., late-onset diagnoses (clinical observations, M.E.). This is thought to be due to a uneven recognition of AEs, a notion underscored by a considerable increase in the AE incidence over the last decennium, with a relatively more pronounced increase in LGI1-antibody encephalitis, which presents more subtly and later in life compared to NMDA-R encephalitis [5].

Thus, the main hypothesis for this study was that some patients attending Swedish memory clinics have an undiagnosed autoantibody-positive AE. Secondarily, such patients were hypothesised to show aberrances in available biomarkers of neuroinflammation and neuronal damage, including CSF cell count [10], neurofilament light (NFL) or tau [11, 12], albumin quotient or kappa-free light-chain-intrathecal fraction [13], enabling a possible pre-selection of individuals at risk of AE that could be further tested.

## Methodology

### Patients and ethical approval

This study was approved by the Swedish Ethical Review Authority, registration number 2011/1987–31 and 2022/03556–01. 1021 patients were retrospectively included from GEDOC, a long-running cohort of patients coming to the memory outpatient clinics at the Karolinska University Hospital, Stockholm, Sweden [14]. Included patients came to the memory outpatient clinic between 2019 and 2023. Patients who were able to do so provided written consent after receiving oral and written information about the study. For patients who could not comprehend the information due to severe cognitive decline, written informed consent was provided by their legal guardian or next of kin.

All patients coming to the Karolinska memory outpatient clinic were asked to participate in the cohort study and approximately 91% of the patients undergoing a lumbar puncture agreed to participation during the inclusion period. The resources available for the cohort includes a biobank of frozen serum and CSF and a database with information on gender, age and diagnosis, as well as scores from neuropsychological testing (*e.g*., MMSE, MoCA, RAVLT), brain MRI, APOE genotyping, and clinical biomarker analyses in CSF and blood (*e.g*., NFL, amyloid β42, amyloid β42/40 quotient, phospho-tau181 and total tau). The sample of patients used in this study was enriched for individuals with available CSF and for whom the kappa-free light-chain-intrathecal fraction (KFLC-IF) had been measured. In the end, 927/1021 (91%) of the study sample had both CSF and KFLC-IF data available.

### Antibody screening and confirmation

The researchers involved in screening were all blinded to diagnostic information, including the fact that one LGI1 patient was present in the dataset. All sera were screened for autoantibodies to CASPR2, LGI1, GABA_B_R, IgLON5, and the NMDAR subunit GRIN1 using in-house live cell-based assays. All assays were repeated at least twice for each serum. In cases where a signal above background was detected consistently in the two replicates, CSF was retrieved and both serum and CSF were sent for external confirmatory testing by commercial cell-based assays at the Karolinska University Hospital Clinical Immunology Laboratory Unit, Stockholm, Sweden, as well as by in-house tissue-based assays performed by the Odense Autoimmune Neurology Group, University of Southern Denmark, Odense, Denmark.

Details on the procedures for all these assays are available in the supplementary materials and methods section. Supplementary Table 1 denotes the plasmids that were specifically produced for this project by VectorBuilder Incorporated (Chicago, IL, USA).

### Statistical analysis

Non-parametric tests, mainly Mann–Whitney U test and Fisher’s exact test, were used for analyses comparing demographic, clinical, and laboratory features of autoantibody-positive and negative patients. Corrections for multiple comparisons are made with false detection rate (FDR) adjustments.

## Results

### Study sample

The study sample consisted of 1021 patients (58% female; median age of 63 (range 34–94)) who were seen at the Karolinska University Hospital memory clinics, Stockholm, from 2019 to 2023. The study sample made up 55% of all patients who underwent a lumbar puncture at the memory clinics in the period, the remaining 45% either lacking available material or declining inclusion in the study. In our study sample, the frequency of a confirmed neurodegenerative dementia diagnosis (Alzheimer´s disease, fronto-temporal, Parkinson´s disease-related, or Lewy-body dementia) in the study sample was 333/1021 (32%), with 525/1021 (51%) only receiving a diagnosis of mild cognitive impairment without any other diagnosis. One patient was diagnosed with LGI1 encephalitis ten years prior. 162 patients suffered from secondary dementia syndromes (*n* = 63) and affective or anxiety-associated disorders (*n* = 53). 46 patients suffered from mild cognitive impairment in addition to other diagnoses, most commonly cerebrovascular disorders, hypertonia, or fibromyalgia, with eight having autoimmune disorders, including multiple sclerosis, psoriasis, and systemic lupus erythematosus (Supplementary Table 2).

### Autoantibody testing

To establish the frequency of neuronal autoantibody seropositivity in the study sample, the patients´ sera were screened in duplicate for CASPR2, GABAB-R, IgLON5, LGI1, and NMDA-R antibodies by live in-house cell-based assays, scored by microscopy (Table 1). For all antigens apart from NMDA-R, samples that showed a consistent positive pattern in both duplicates were selected for confirmation: 6 were positive for CASPR2, 2 for GABA_B_-R, and 2 for LGI1 autoantibodies. As NMDA-R screening was done in serum for logistical reasons, despite higher sensitivity and specificity for NMDA-R autoantibodies in CSF [15], a total of 27 CSF samples from patients whose sera showed any signs of NMDA-R antibody positivity were also tested for antibodies in CSF, of whom six were also CSF-positive. Two patients with NMDA-R antibody-positive serum samples were excluded, as no CSF was available for testing.

**Table 1:** Number of positive samples of all tested samples

Antigen	CASPR2	GABAB-R	IgLON5	LGI1	NMDA-R
Live CBA serum	6 of 1021	2 of 1021	0 of 1021	2 of 1021	27 of 1021
Live CBA CSF	3 of 6	2 of 2	0	0 of 2	6 of 25
Fixed CBA serum	2 of 6	0 of 2	0	1 of 2	2 of 6**
Fixed CBA CSF	0 of 4	0 of 2	0	0 of 2	1 of 6
TBA serum	6 of 6	2 of 2	0	1 of 2	5 of 6
TBA CSF	4 of 6	0 of 2	0	1 of 2	5 of 6
Confirmed	5* of 1021	2 of 1021	0 of 1021	1 of 1021	3 of 1021***

All samples were screened with live-CBA tests in serum. Only samples positive at this screening stage were tested further. For NMDA-R, only samples also positive in CSF were tested further

*6th patient had an end-point titer of 1/100 in serum in the live-CBA and was negative in all other tests, apart from the serum TBA. This was considered below threshold, and the patient was excluded

**A third patient was positive, but showed an ANA antibody pattern in the clinical assay and was therefore excluded

***The two additionally excluded patients had end-point titres of 1:20, were borderline-positive in the live CBA in CSF and tested negative in the commercial assays

**Fig. 1:**
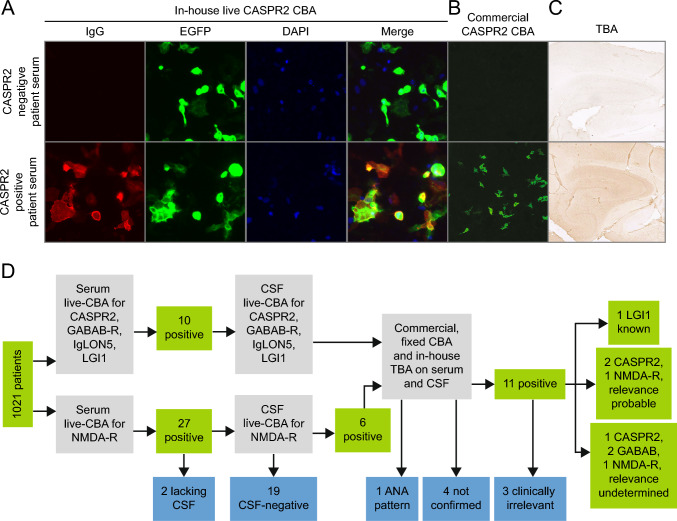
Example of one CASPR2-negative and one CASPR2-positive serum sample in the in-house live cell-based assay (CBA, **A**), the commercial CBA (**B**) and the tissue-based assay (TBA, **C**), i.e., a horse peroxidase-stained rodent brain section. For the in-house live CBA, green depicts enhanced green fluorescent protein (eGFP) and red depicts IgG staining, whereas for the commercial assay, green depicts IgG staining. DAPI = 4′,6-diamidino-2-phenylindole, a nucleic acid dye depicting cell nuclei. **D** Flowchart of screening setup

Serum and CSF samples from patients identified as antibody-positive by live in-house CBA (6 CASPR2, 2 GABA_B_-R, 2 LGI1, and 6 NMDA-R antibody-positive samples) were further evaluated by live-CBA end-point titrations, as well as confirmatory testing with commercial fixed CBAs and by IHC on rodent brain sections (Fig. 1, Table 1). A patient was considered antibody positive if their serum was positive by in-house live CBA and either their serum or CSF were positive by at least two other tests. For NMDA-R positive patients, the presence of CSF autoantibodies in two tests wa required, to ensure test specificity, as low-level serum NMDA-R antibodies have been seen in patients with affective disorders, Parkinson´s disease, and stroke; all common diagnoses in the cohort [16]. This procedure identified 11 patients that harbored autoantibodies of possible clinical significance against CASPR2 [5], GABAB-R [2], LGI1 [1], and NMDA-R ([3]; Tables 1 and 2). No patients were positive for IgLON5 autoantibodies.

**Table 2:** Cases with positive test results

Antigen		CASPR2					GABAB-R		NMDA-R			LGI1
Case		1	2	3	4	5	6	7	8	9	10	11
Live CBA EPT	Serum	1/3200	1/16400	1/1600	1/100	1/400	> 1/320	1/160	1/320	1/160	1/20	1/80
	CSF	1/16	1/32	1/2	–	–	1/4	1/1	> 1/16	1/4	1/2	–
Fixed CBA	Serum	+	+	–	–	–	–	–	+	+	–	+
	CSF	NA	NA	–	–	–	–	–	+	–	–	–
TBA	Serum	+	+	+	+	+	+	+	+	–	+	+
	CSF	+	+	+	+	–	–	–	–	+	+	+
Antibody of potential clinical relevance		TRUE	TRUE	TRUE	FALSE	FALSE	TRUE	TRUE	TRUE	TRUE	FALSE	TRUE

*EPT* end-point titer, *CBA* cell-based assay, *TBA* tissue-based assay

### Clinical characterization of antibody-positive cases

**Fig. 2:**
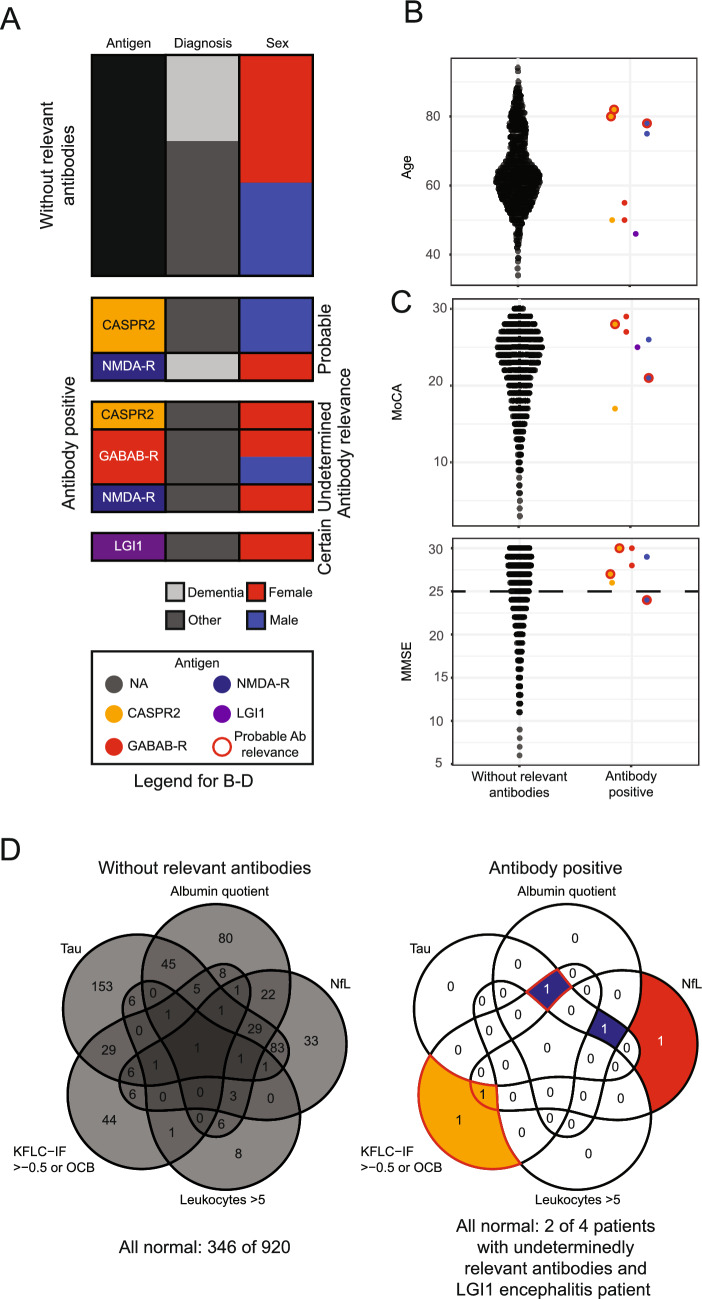
Characteristics of identified patients. **A**: Sex ratio and fraction of dementia diagnoses in the antibody-negative and positive patients, further stratified by subgroup. Differences are not significant in either case. **B**: Age distribution among antibody-negative and positive patients. **C**: MoCA/MMSE test results assessed close to the time of the lumbar puncture. Differences are not significant in any of the comparisons. Dashed line in MMSE graph indicates threshold for normal tests. For MoCA, the threshold varies depending on age and educational background. **D**: Venn diagram showing the distribution of the five laboratory tests most directly reflecting neuroinflammation. KFLC-IF = kappa-free light-chain-intrathecal fraction. OCB: CSF-specific oligoclonal bands in gel electrophoresis. NB! Single CSF-specific bands are also included as OCB. All three individuals with high likelihood of autoantibody relevance were either borderline-positive for KFLC-IF or OCB (False detection rate-corrected Fisher´s exact test p = 0.01 compared to antibody-negative patients). No other individual markers differed statistically. Due to the low number of autoantibody-positive cases and their apparent Venn diagram heterogeneity, no formal statistical analysis of differences in the overarching Venn diagram distribution was attempted. In **B**–**D**, the red borders indicate that the denoted patient harbors antibodies of probable significance

Next, the antibody-positive cases were clinically characterized (Fig. 2, Table 3 and supplementary Table 3). One patient was identified with LGI1 autoantibodies. They had been diagnosed with and treated for LGI1 encephalitis 10 years prior. As the testing was performed blind to clinical information, this patient was not identified until the screening was complete. Of note, there were no other patients in the study sample that had a previous diagnosis of AE. The remaining ten antibody-positive patients had not been investigated for AE prior to or during the memory clinic diagnostic work. When investigating the clinical history, three antibody-positive results were deemed irrelevant, with low-titer antibodies to CASPR2 and NMDA-R. The patients were characterized by slow-onset memory impairment, all fulfilled Alzheimer dementia criteria, had low antibody levels, low inter-test reproducibility, and lacked associated focal neurological and psychiatric symptoms.

**Table 3:** Clinical characterization of patients with definite, likely or uncertain autoantibody relevance

Antigen	CASPR2			GABAB-R		NMDA-R		LGI1
Case	1	2	3	6	7	8	9	11
Antibody relevance	Probable	Probable	Undetermined	Undetermined	Undetermined	Probable	Undetermined	Definite
Diagnosis at memory clinic	MCI	MCI	MCI	MCI	MCI	Alzheimer´s disease	MCI	LGI1 encephalitis
Age at memory clinic visit	> 75	> 75	< 56	< 56	< 56	> 75	> 75	< 56
Time since onset	8 month	11 years	7 month	3 years	15 years	3 years	2 years	10 years
Healthcare delay	6 month	5 years	3 month	6 months	15 years	1 year	4 month	10 years
Neuropsychology	NA	Aberrant	Aberrant	Aberrant	Normal	Aberrant	Aberrant	NA
MoCA	28	NA	17	29	27	21	26	25
MMSE	30	27	26	28	30	24	29	NA
Psychiatric findings	No	No	Auditory hallucinosis, affect lability, sexual misconduct	No	No	Obcessive compulsions	Affect lability	No
Neurologicalfindings	Neuro-myotonia**	Seizures, neuropathic pain	Memory gaps	Upper extremity muscle rigidity	Normal	Normal	Normal	Normal*
Malignancy	No	No	No	No	Cognitive symptoms since breast cancer	Ataxia + Yo + Hu-antibodies + breast cancer diagnosis 3 years later	Prostate cancer 2 years later	No

*MCI* mild cognitive impairment. *MoCA* Montreal cognitive assessment, *MMSE* mini-mental state examination

*Patient did have seizures at diagnosis 10 years prior. **Neuromyotonia documented at memory clinic but only diagnosed post hoc

Three of the remaining seven individuals had phenotypes indicative of an ongoing autoimmune or paraneoplastic encephalitis, respectively. Two of these were elderly male patients with serum CASPR2 autoantibody end-point titres of 1 in 3000 and 1 in 15,000 and concordant positivity across all antibody tests. One CASPR2 antibody-positive patient had subacute onset of cognitive decline, concurrent with onset of epilepsy and neuropathic pain. This patient was offered treatment but declined. The second CASPR2 antibody-positive patient had a slower-onset of cognitive decline, including a 5-year healthcare delay, concurrent with neuromyotonia, which subsequently responded to treatment (methylprednisolone, plasma exchange and rituximab). A third patient, also fulfilling Alzheimer´s dementia criteria, had NMDAR antibodies in serum and CSF and tested positive in all tests. This patient had a sudden worsening of previous cognitive decline. In the following years, this patient developed ataxia and breast cancer with Hu and Yo antibodies. For the remaining four patients, it could not be determined if the antibodies were a sign of ongoing, low-grade inflammation, a remnant of previous encephalitis, or of no pathogenic relevance. These patients had low-to-medium end-point antibody titres to CASPR2, GABA_B_-R, and NMDA-R with acceptable inter-test reproducibility (Table 2). As an example of their clinical phenotype, a middle-aged female patient with GABA_B_-R antibodies in serum and CSF presented with cognitive decline concurrently with a breast cancer diagnosis a few years earlier. Having since improved, her symptoms were diagnosed as relating to an "exhaustion disorder" and she also received an autism diagnosis, despite lack of information about childhood autism symptoms.

### Brain imaging and laboratory test results for positive cases and controls

Next, paraclinical results for the seven patients with antibodies of probable or undetermined significance were investigated, aiming to identify biomarkers to evaluate the risk of antibody-mediated AE.

No patient showed signs of active neuroinflammation on brain magnetic resonance tomography (MRT), such as mediotemporal T2-weighted fluid-attenuated inversion recovery (FLAIR) hyperintensities. The CASPR2 antibody-positive patient with long healthcare delay and neuromyotonia showed significant age-adjusted medial temporal lobe atrophy (MTA 2) [14], which was also the case for the previously diagnosed LGI1 patient. Three patients had mild-to-moderate global atrophy (GCA 2). Four patients, all above 75 years of age, showed signs of small vessel disease due to the presence of deep cerebral microhaemorrhages and lacunar infarcts. This was also the case for the patient with NMDA-R antibodies of undetermined relevance, who had extensive white matter changes (Fazekas 3, Table 4). Compared to previous results from the Karolinska memory clinic cohort [14], these imaging findings are on average somewhat less pathological, but with 75% of the overall cohort showing normal results as measured by MTA [14], this absence of degenerative findings also does not separate the antibody-positive patients from their antibody-negative peers.

**Table 4:** Paraclinical test results, and fulfillment of Graus possible autoimmune encephalitis criteria

Antigen		CASPR2			GABAB-R		NMDA-R		LGI1
Case		1	2	3	6	7	8	9	11
Antibody relevance	Probable	TRUE	TRUE	–	–	–	TRUE	–	Previously diagnosed
	Undetermined	–	–	TRUE	TRUE	TRUE	–	TRUE	
Brain MRT	Signs of neuroinflammation	No	No	No	No	No	No	NA	No
	Fazekas, average	1	1	0.5	NA	1	1	3	1
	MTA, average	1.5	2	0.5	NA	0	1	0.5	2
	GCA, average	1	1.5	0.5	NA	0.5	1.5	1.5	0.5
	Old cerebrovascular lesions (all small)	1 right cere-bellar	1–2 lacunar	none	none	none	2 micro-hemorrhages*	1 left medial frontal lobe	none
CSF test results	Leukocytes/microliter	0	4	0	0	1	3	0	0
	Increased albumin quotient	No	No	No	No	No	Yes	Yes	No
	Increased KFLC-IF or OCB	Yes	Yes	No	No	No	Yes	No	No
	Increased neurofilament light	No	Yes	No	No	Yes	No	Yes	No
	Increased total tau	No	No	No	No	No	Yes	Yes	No
	Increased phosphorylated tau	No	No	No	No	No	Yes	Yes	No
	Decreased β-amyloid 42	Yes	No	No	No	No	No	No	No
	Decreased β-amyloid 40/42 quotient	Yes	No	No	No	No	Yes	Yes	No
	STAM3^m^P score**	1	1	1	1	1	1	0	1

*KFLC-IF* kappa-free light-chain-intrathecal fraction, *OCB* selectively observed oligoclonal bands in CSF compared to serum on isoelectric focusing, *MRT* magnetic resonance tomography, *Fazekas* white mater hyperintensity load score (0–3), indicating grade of small vessel disease. *MTA* = medial temporal lobe atrophy score (0–4, 0 and 1 normal), *GCA* global cortical atrophy scale (0–4). *See discussion

*One occipital, one cerebellar microhemorrhage

**Features of the scale are: Seizures at presentation, Disease-associated tumor, Age-at-symptom onset < 50 years, Mania, Movement abnormalities, MRI suggestive of AE, Dementia within 3 months after symptom onset, Pleocytosis, ≥ 10 cells/μL in CSF

We then investigated laboratory test results linked to possible neuroinflammation, including CSF leukocyte count, albumin quotient, CSF-specific oligoclonal electrophoresis bands (OCB) and kappa-free light-chain-intrathecal fraction (KFLC-IF), CSF neurofilament light (CSF-NfL, marker of axonal damage), and total tau (marker of neuronal cell body damage). None of the patients had leukocytes above 5 per microliter. The three patients with autoantibodies of probable relevance had suggestions of intrathecal antibody production: one with a borderline KFLC-IF, one with positive oligoclonal bands (with no KFLC-IF analysis), and a third with normal KFLC-IF but one CSF-specific band (Fisher´s test p value 0.01 after false detection rate adjustment for multiple comparisons). One of the CASPR2 antibody-positive patients also showed increased CSF-NfL with the NMDA-R antibody-positive patient showing affected albumin quotient and total tau. Of the remaining four patients, two had normal test results, and two had increased CSF-NfL. Apart from the intrathecal antibody production finding, none of these results were significantly different from the rest of the study sample, considering either each individual marker or calculating the likelihood of all being normal (Fisher´s exact test p value of 1 for albumin quotient, CSF-NfL, total tau, and all 5 tests normal, with false detection rate adjustment for multiple comparisons, Table 4, Supplementary Table 3).

Next, a wider analysis of the remaining 23 laboratory test results widely available for the study sample was examined. This included markers of neurodegeneration such as CSF ß-amyloid 42, but also standard blood tests, such as hemoglobin, electrolytes, and markers of kidney and liver function. No additional statistical differences were identified, either when each variable was considered separately (Supplementary Table 3).

Finally, a random forest-based classification algorithm was used to identify if combinations of the CSF leukocyte count, albumin quotient, intrathecal antibody production markers, CSF-NfL, total tau, and the additional 23 variables could discriminate the antibody-positive patients from the rest. This was not the case (data not shown).

## Discussion

In this work, we identify eight individuals with neuronal autoantibodies among 1021 individuals undergoing dementia diagnostics at the Karolinska University Hospital, seven of whom had not been previously diagnosed with AE. Of these, three had a convincing clinical phenotype of autoimmune or paraneoplastic encephalitis and are called “likely AE/PE” below. Notably, these three likely AE/PE patients did not fulfill the time criterion of possible autoimmune encephalitis, as postulated by Graus and colleagues in 2016. This criterion is, however, often not formally fulfilled in late-onset AE; patients suffering from CASPR2 encephalitis e.g., often have 1 year from first symptom to diagnosis, due to an insidious onset, leading to a combination of patient´s and doctor´s delay [17]. However, the three patients had neurological symptoms of relevance: new-onset epilepsy/neuropathic pain, neuromyotonia, or rapid-onset ataxia with breast cancer and associated paraneoplastic antibodies. How common these symptoms were in the overall study sample is not possible to judge, as data were only extracted from the patient files of the antibody-positive individuals due to ethical constraints. For the remaining four patients, the clinical relevance of their antibody positivity was not possible to determine with available information and test results.

Not unexpectedly, the three cases of likely AE showed subtle signs of intrathecal antibody production, compared to 10% in the overall study sample. Oligoclonal CSF-specific bands (OCB) has been previously associated with autoimmune encephalitis, but is not seen in more than half of patients in other studies [20]. Notably, KFLC-IF seemed less sensitive than the presence of individual CSF-specific bands: of the two patients for whom KFLC-IF measurements were available, one was borderline positive, and the other was negative, but showed one CSF-specific band. The third patient had oligoclonal bands. Given that the CSF output of antibodies often is low but highly antigen-specific in later-onset AE and that clonal sharing between blood and CSF seems restricted [21, 22], it is biologically plausible that even single, CSF-specific electrophoresis bands are of relevance in this disorder. It is likely that the frequency of CSF-specific electrophoresis bands in the study sample overall is an underestimate for three reasons: the common test for intrathecal antibody production at the Karolinska memory clinics is KFLC-IF, information about oligoclonal bands is not systematically reported in the database, and standard reporting does not call one CSF-specific electrophoresis band positive. Still, it is possible that a wider definition of intrathecal antibody production in the context of AE screening, including single CSF-specific electrophoresis bands, might yield a high sensitivity for this widely available test, but it would suffer from low specificity, limiting clinical utility.

Four of the seven patients showed normal levels of CSF-NfL, of whom two were likely AE patients. Studies on CSF-NfL in AE show that 50% of patients with antibodies to LGI1 and as many as 80% of idiopathic or teratoma-associated NMDA-R encephalitis patients have normal levels of CSF-NfL [12]. Further, as CSF-NfL was increased in 20% of the antibody-negative patients, this marker seems unsuitable as a biomarker for AE in this context. Our low frequency of likely AE stands in stark contrast to a recent study by van Steenhoven et al., who report an AE frequency of 40% among rapidly developing dementia patients [18]. This indicates that our outpatient setting and referral system selects for slower-onset cases. Therefore, the risk of overlooking an AE diagnosis in the memory clinic is comparatively low. However, there are also indications that our study might have underestimated the frequency of AE in the sample; with screening efforts directed only at antibodies against five specific AE antigens, it is possible that patients with antibodies against other antigens were missed. Indeed, work by Giannocaro et al. [23], patients diagnosed with neurodegenerative conditions were shown to harbor autoantibodies to the glycine and GAB_A_A receptors, in addition to LGI1, CASPR2, and GABABR. Our study also did not systematically investigate paraneoplastic antibodies. An approach combining live cell-based assays for the most common antigens with tissue-based screening would, therefore, have been preferable. Still, it is likely that the low AE frequency in our data primarily reflects a selection of slower-onset cases to this outpatient clinic; as many patients undergo brain MRI prior to referral to the memory clinic, individuals with typical signs of encephalitis might instead be referred to a neurology center focusing on neuroinflammation.

There are further reasons that our results cannot be generalized to estimate the prevalence of AE/PE in memory clinics on a national or international level. 1. The Karolinska Memory clinic catchment area for individuals with early onset memory impairment is larger than for dementia of any cause, skewing the study sample toward younger individuals (4% of the cohort are below 50 years of age). 2. As our study sample is enriched for individuals with KFLC-IF measurements, it might be biased toward more severe cases; indeed, in work by Rosenberg et al. [14] on the same Karolinska memory clinic cohort, the rate of dementia was 23%, compared to 33% in our study. With all these caveats in mind, the current AE incidence estimate is 3 per million person-years in Sweden [9], or 30 new cases per year. If the true prevalence of undiagnosed AE for memory clinic patients would be conservatively estimated to 1/10 of our study result, or 0.03%, with 75 000 unique memory clinic investigations per year [24], these undiagnosed and untreated AE patients would nearly double the total AE cases. On the one hand, this underscores the potential gains of finding an acceptably specific way to identify individuals with possible AE for further testing. On the other hand, it underlines that antibody tests are not suitable for unselective screening; societal costs aside, with such a low pre-test probability, a large volume of false-positive test results would be generated for every true-positive result, with associated harm to patients. Furthermore, given the known negative associations between treatment response and time from onset as well as memory impairment [25], it is not even certain that all patients with a confirmed autoimmune encephalitis in the memory clinic setting would benefit from immunotherapy. Thus, an ideal AE biomarker in a memory clinic setting would not only sensitively identify potential AE cases, but also stratify these patients based on likely treatment responsiveness.

Recently, the clinical STAM3^m^P score was developed to identify patients with a treatable cause of rapidly developing dementia [19]. When using this score on our seven antibody-positive cases, the three cases of likely AE only get one point each, as do three of the four remaining antibody-positive patients (Table 4). Even if this is applied slightly outside of its original context (many of the patients did in fact not fulfill rapidly progressing dementia criteria) and even as the cohort as a whole has not been investigated, this indicates that this score, in combination with careful clinical investigations, might in fact be sufficiently sensitive to be useful as a pre-selection tool for treatable dementia, and therefore indirectly AE, also in a Swedish memory clinic outpatient setting.

In conclusion, autoimmune encephalitis is a cause to consider for patients with memory impairment in combination with new, unexplained neurological symptoms. With a low frequency and insufficient sensitivity and specificity of widely used markers of neuroinflammation and autoantibody tests, unselective screening for AE for individuals with memory impairment cannot be recommended. Our data instead indicates that a careful clinical approach, supported, *e.g.,* by the STAM3^m^P score, might acceptably limit the number of missed AE treatment opportunities in the memory clinic. Still, identifying feasible biomarkers for systematic AE risk stratification and treatability in memory clinics remains a goal for future studies.

## Supplementary Information

Below is the link to the electronic supplementary material.

Supplementary file1 (DOCX 23 KB)

Supplementary file2 (XLSX 9 KB)

Supplementary file3 (XLSX 9 KB)

Supplementary file4 (XLSX 11 KB)

## Data Availability

For each sample and antigen, raw.nd2 image files are available for download at 10.48723/m0cq-4k94. This repository includes a file linking individual images with demographic and clinical data shown in Fig. 2 and information on whether they were considered positive with each test method. These data have been rated as of potentially sensitive nature by the Swedish National Data Service (SNDS) and thus, access requests will go through a review process determined by the SNDS.

## References

[1] Varley J, Taylor J, Irani SR (2017) Autoantibody-mediated diseases of the CNS: structure, dysfunction and therapy. Neuropharmacology. 10.1016/j.neuropharm.2017.04.046. (**PubMed PMID: 28476644**) 28476644 10.1016/j.neuropharm.2017.04.046

[2] Sillevis Smitt P, Kinoshita A, De Leeuw B, Moll W, Coesmans M, Jaarsma D et al (2000) Paraneoplastic cerebellar ataxia due to autoantibodies against a glutamate receptor. N Engl J Med 342(1):21–27. 10.1056/NEJM200001063420104. (**PubMed PMID: 10620645**) 10620645 10.1056/NEJM200001063420104

[3] Dalmau J, Tüzün E, Wu H, Masjuan J, Rossi JE, Voloschin A et al (2007) Paraneoplastic anti–N-methyl-D-aspartate receptor encephalitis associated with ovarian teratoma. Ann Neurol 61(1):25–36. 10.1002/ana.21050 17262855 10.1002/ana.21050 PMC2430743

[4] Vincent A, Buckley C, Schott JM, Baker I, Dewar BK, Detert N et al (2004) Potassium channel antibody-associated encephalopathy: a potentially immunotherapy-responsive form of limbic encephalitis. Brain 127(Pt 3):701–712. 10.1093/brain/awh077. (**PubMed PMID: 14960497**) 14960497 10.1093/brain/awh077

[5] Hébert J, Riche B, Vogrig A, Muñiz-Castrillo S, Joubert B, Picard G et al (2020) Epidemiology of paraneoplastic neurologic syndromes and autoimmune encephalitides in France. Neurol Neuroimmunol Neuroinflamm 7(6):e883. 10.1212/NXI.0000000000000883. (**PubMedPMID:32847939; PubMedCentralPMCID:PMC7455315**) 32847939 10.1212/NXI.0000000000000883 PMC7455315

[6] Varley JA, Strippel C, Handel A, Irani SR (2023) Autoimmune encephalitis: recent clinical and biological advances. J Neurol 270(8):4118–4131. 10.1007/s00415-023-11685-3 37115360 10.1007/s00415-023-11685-3 PMC10345035

[7] Bastiaansen AEM, van Steenhoven RW, de Bruijn MAAM, Crijnen YS, van Sonderen A, van Coevorden-Hameete MH et al (2021) Autoimmune encephalitis resembling dementia syndromes. Neurol Neuroimmunol Neuroinflamm 8(5):e1039. 10.1212/NXI.0000000000001039. (**PubMedPMID:34341093; PubMedCentralPMCID:PMC8362342**) 34341093 10.1212/NXI.0000000000001039 PMC8362342

[8] Bastiaansen AEM, van Steenhoven RW, Te Vaarwerk ES, van der Flier WM, Teunissen C, de Graaff E et al (2023) Antibodies associated with autoimmune encephalitis in patients with presumed neurodegenerative dementia. Neurol Neuroimmunol Neuroinflamm 10(5):e200137. 10.1212/NXI.0000000000200137. (**PubMedPMID:37311646; PubMedCentralPMCID:PMC10265404**) 37311646 10.1212/NXI.0000000000200137 PMC10265404

[9] Kosek S, Persson B, Rodrigues R, Malmeström C, Punga AR, Burman J (2023) Antibody-positive autoimmune encephalitis and paraneoplastic neurological syndrome: epidemiology and outcome of neuronal antibody testing in Sweden. Acta Neurol Scand 10(2023):e6993615. 10.1155/2023/6993615

[10] Graus F, Titulaer MJ, Balu R, Benseler S, Bien CG, Cellucci T et al (2016) A clinical approach to diagnosis of autoimmune encephalitis. Lancet Neurol 15(4):391–404. 10.1016/S1474-4422(15)00401-9. (**PubMedPMID:26906964; PubMedCentralPMCID:PMC5066574**) 26906964 10.1016/S1474-4422(15)00401-9 PMC5066574

[11] Körtvelyessy P, Prüss H, Thurner L, Maetzler W, Vittore-Welliong D, Schultze-Amberger J et al (2018) Biomarkers of neurodegeneration in autoimmune-mediated encephalitis. Front Neurol 19:9. 10.3389/fneur.2018.00668 10.3389/fneur.2018.00668 PMC6156245 30283395

[12] Nissen MS, Ryding M, Nilsson AC, Madsen JS, Olsen DA, Halekoh U et al (2021) CSF-neurofilament light chain levels in NMDAR and LGI1 encephalitis: a national cohort study. Front Immunol 12:719432. 10.3389/fimmu.2021.719432 34975832 10.3389/fimmu.2021.719432 PMC8716734

[13] Bertram D, Tsaktanis T, Berthele A, Korn T (2023) The role of intrathecal free light chains kappa for the detection of autoimmune encephalitis in subacute onset neuropsychiatric syndromes. Sci Rep 13(1):17224. 10.1038/s41598-023-44427-6 37821561 10.1038/s41598-023-44427-6 PMC10567819

[14] Rosenberg A, Öhlund-Wistbacka U, Hall A, Bonnard A, Hagman G, Rydén M et al (2022) β-amyloid, tau, neurodegeneration classification and eligibility for anti-amyloid treatment in a memory clinic population. Neurology 99(19):e2102–e2113. 10.1212/WNL.0000000000201043 36130840 10.1212/WNL.0000000000201043 PMC9651451

[15] Gresa-Arribas N, Titulaer MJ, Torrents A, Aguilar E, McCracken L, Leypoldt F et al (2014) Antibody titres at diagnosis and during follow-up of anti-NMDA receptor encephalitis: a retrospective study. Lancet Neurol 13(2):167–177. 10.1016/S1474-4422(13)70282-5 24360484 10.1016/S1474-4422(13)70282-5 PMC4006368

[16] Dahm L, Ott C, Steiner J, Stepniak B, Teegen B, Saschenbrecker S et al (2014) Seroprevalence of autoantibodies against brain antigens in health and disease. Ann Neurol 76(1):82–94. 10.1002/ana.24189 24853231 10.1002/ana.24189

[17] Distinct phenotypes in a cohort of anti-CASPR2 associated neurological syndromes. Clin Neurol Neurosurg, 2023;234:107994. 10.1016/j.clineuro.2023.107994 10.1016/j.clineuro.2023.107994 37797365

[18] van Steenhoven R, Bastiaansen D, de Vries J, de Jong FJ, Satyadev N, Brenner J et al (2025) Autoimmune encephalitis is the most common treatment-responsive cause of rapidly progressive dementia; a large dutch prospective cohort study. (PL4.002). Neurology 104(7_Supplement_1):3386. 10.1212/WNL.0000000000210982

[19] van Steenhoven RW, Satyadev N, Bastiaansen AEM, Piura YD, Kerstens J, Crijnen YS et al (2026) Early recognition of treatment-responsive rapidly progressive dementia: the modified STAM3mP score. Ann Clin Transl Neurol. 10.1002/acn3.70434 42203753 10.1002/acn3.70434 PMC13394507

[20] Zrzavy T, Höftberger R, Wimmer I, Berger T, Rommer P, Macher S (2021) Longitudinal CSF findings in autoimmune encephalitis-a monocentric cohort study. Front Immunol 12:646940. 10.3389/fimmu.2021.646940. (**PubMedPMID:33828556; PubMedCentralPMCID:PMC8019787**) 33828556 10.3389/fimmu.2021.646940 PMC8019787

[21] Theorell J, Harrison R, Williams R, Raybould MIJ, Zhao M, Fox H et al (2024) Ultrahigh frequencies of peripherally matured LGI1- and CASPR2-reactive B cells characterize the cerebrospinal fluid in autoimmune encephalitis. Proc Natl Acad Sci 121(7):e2311049121. 10.1073/pnas.2311049121 38319973 10.1073/pnas.2311049121 PMC10873633

[22] Esser D, Müller-Miny L, Heming M, Paunovic M, van Duijn M, Abrante Cabrera L et al (2025) Activated αβ T and reduced mucosa-associated invariant T cells in LGI1- and CASPR2-encephalitis. Brain 148(9):3170–3183. 10.1093/brain/awaf096. (**PubMedPMID:40094812; PubMedCentralPMCID:PMC12404778**) 40094812 10.1093/brain/awaf096 PMC12404778

[23] Giannoccaro MP, Gastaldi M, Rizzo G, Jacobson L, Vacchiano V, Perini G et al (2021) Antibodies to neuronal surface antigens in patients with a clinical diagnosis of neurodegenerative disorder. Brain Behav Immun 96:106–112. 10.1016/j.bbi.2021.05.017. (**PubMed PMID: 34022370**) 34022370 10.1016/j.bbi.2021.05.017

[24] Mattke S, Gustavsson A, Jacobs L, Kern S, Palmqvist S, Eriksdotter M et al (2024) Estimates of current capacity for diagnosing alzheimer’s disease in sweden and the need to expand specialist numbers. J Prev Alzheimers Dis 11(1):155–161. 10.14283/jpad.2023.94 38230728 10.14283/jpad.2023.94 PMC10995070

[25] Thompson J, Bi M, Murchison AG, Makuch M, Bien CG, Chu K et al (2018) The importance of early immunotherapy in patients with faciobrachial dystonic seizures. Brain 141(2):348–356. 10.1093/brain/awx323 29272336 10.1093/brain/awx323 PMC5837230

