# Supplementary material for: Screening 1021 Swedish memory clinic visitors for autoantibody-mediated encephalitis
Source: J Neurol. 2026 Jun 20;273(7):408. doi: 10.1007/s00415-026-13953-4 (PMC13283170; doi:10.1007/s00415-026-13953-4)
Supplement: Supplementary file 1 — Supplementary file1 (DOCX 23 KB) [file 415_2026_13953_MOESM1_ESM.docx]

- 1. **Plasmids**

Plasmids for CASPR2, GABA_B_R and IgLON5 were designed using VectorBuilder (Chicago, IL, USA) (Supplementary table 1). Membrane bound LGI1 fusion protein and NMDAR (GRIN1) plasmids were kindly provided by the Oxford Autoimmune Neurology Diagnostic Laboratory, University of Oxford, UK, (1, 2). For CASPR2, IgLON5 and the LGI1 fusion protein, eGFP was fused to the intracellular c-terminus. For the GABA_B_R assay, cells were transfected with a 5:2 proportion of the GABA_B_R1 and GABA_B_R2 plasmids. The GABA_B_-R1 and -R2 proteins were fused to intracellular, c-terminal FLAG tags, that were not used in this project. For the NMDAR antibody detection, a plasmid containing the native GRIN1 construct was used without any GRIN2 construct. The plasmids were either bought ready to use from VectorBuilder (CASPR2, and IgLON5) or expanded and extracted from bacteria using EndoFree MaxiPrep kits (Qiagen, Venlo, the Netherlands). The competent bacteria transformations were performed by VectorBuilder for GABA_B_R and in-house for GRIN1 and LGI1 constructs.

- 1. **Live cell-based assay (CBA)**

A detailed CBA protocol with modifications for each antigen can be downloaded at https://github.com/jtheorell/Laboratory-protocols. It is based on published protocols (Irani 2010) with a few modifications. Instead of conventional HEK-293 cells, a HEK-293 derived cell line expressing the macrophage scavenger receptor, called GripTite™ 293 MSR cells were used, as these more strongly adhere to plastic. Lipofectamine (Invitrogen) was used as a transfection agent. For GRIN1, the NMDA-R inhibitor MK801 was added eight hours after transfection with a final concentration of 30 μM. Time from transfection to screening was 48 hours for all antigens apart from GRIN1, where 24 hours was used.

- 1. **Screening procedure**

For screening, sera were diluted 1:100 for CASPR2 and 1:20 for the remaining antigens. Negative and positive controls from the Autoimmune Neurology Diagnostic Laboratory, University of Oxford, UK (all but IgLON5) and the Autoimmune Neurology Group, University of Southern Denmark, Odense, Denmark (IgLON5) were included in all plates. To ensure a clear positive signal, the positive controls were used at concentrations at least 4 times higher than their individual end-point titres. All screening was performed in duplicate. CSF was screened undiluted. For titrations of positive samples, the end-point titre was defined as the highest dilution giving a positive score. For all antigens apart from GRIN1, the secondary antibody was a goat anti-human cross-absorbed IgG (H+L) conjugated to Alexa fluor 568 (Invitrogen). For NMDA-R, the secondary antibody was a goat anti-human Fc cross-absorbed IgG (Invitrogen), followed by a tertiary donkey-anti-goat Fc cross-adsorbed IgG conjugated to Alexa fluor 568 (Invitrogen). Finally, all wells were coated with a 4′,6-diamidino-2-phenylindole (DAPI)-containing mounting medium (ThermoFisher), staining nuclei blue.

Microscopy imaging of live cell-based assays was performed using a Nikon Ti2 inverted widefield/spinning disk CREST v3 microscope. This system was equipped with a fully motorized stage, a 10x (NA 0.45), or 20x air objective (NA 0.75), and a Kinetix sCMOS camera (> 95% quantum efficiency, 6.5 μm pixel size, 2720 × 2720-pixel field of view), including 1. Images from three random positions in the central area of each well selected by the microscope software were captured per well. The files were saved in .nd2 format, with a 16-bit intensity range with identical intensities for all images within each plate. The imaging was fully automated, including finding the focal plane. Images taken with this robotised microscopy approach were scored by visual inspection.

- 1. **Experimental setup and analysis of confirmatory tissue-based assays**

For tissue-based screening, adult Sprague-Dawley rats were euthanized and brains removed under aseptic conditions. Brains were immediately submersed in cold 4% paraformaldehyde in Phosphate-buffered saline (PBS), for 1 hour. After this, brains were transferred to 40% sucrose in PBS for 48 hours at 4^o^C, for cryoprotection. Brains were separated into hemispheres and individual hemispheres were encapsulated in cryoprotection media (Cryoembed, Leica microsystems) and snap frozen in ice-cold isopentane. Individual capsules were kept at -80^o^C until sectioning. Brains were cut into 6 micrometer tissue sections and placed on microscope slides (Superfrost plus, Thermo-Fischer). Tissue sections were kept at -20^o^C until used. For immunohistochemistry, sections were thawed to room temperature and washed in tris buffered saline (TBS), followed by incubation with H_2_O_2_ in TBS for 30 minutes, then washed in TBS and blocked with blocking buffer containing 2% bovine serum albumin and 5% donkey serum in TBS for 30 min. After additional washing, slides were incubated with serum (1:50) or CSF (1:2) in blocking buffer overnight at 4^o^C. The next day, slides where washed in TBS and incubated with secondary antibody (donkey anti-human IgG, 1:2000, Thermo-Fisher) for 1 h at room temperature. Slides were developed using a standard diaminobenzidine (DAB) protocol and coverslipped in dibutylphthalate polystyrene xylene mounting media (Thermo-Fisher). Slides were visualized using bright-field microscopy and scored visually. As positive controls, IgG purified from the serum of patients with NMDAR-, IgLON5- and LGI1-antibody encephalitis with definite AE and a classic clinical phenotype, was used.

- 1. **Experimental setup and analysis of confirmatory, commercial cell-based assays**

Confirmatory analysis of neuronal surface antibodies was performed using commercial fixed cell-based assays (Euroimmun, Lübeck, Germany) in accordance with the manufacturer’s instructions. CSF samples were tested neat, and serum samples at 1:10 dilution. Fluorescence signals were assessed by indirect immunofluorescence microscopy and graded as negative or positive. Each run included in-house positive controls as well as manufacturer-provided positive and negative controls.

Bibliography

1. S. R. Irani, *et al.*, N-methyl-D-aspartate antibody encephalitis: temporal progression of clinical and paraclinical observations in a predominantly non-paraneoplastic disorder of both sexes. *Brain* **133**, 1655–1667 (2010).

2. S. R. Irani, *et al.*, Antibodies to Kv1 potassium channel-complex proteins leucine-rich, glioma inactivated 1 protein and contactin-associated protein-2 in limbic encephalitis, Morvan’s syndrome and acquired neuromyotonia. *Brain* **133**, 2734–2748 (2010).
